# CtBP1 promotes tumour‐associated macrophage infiltration and progression in non–small‐cell lung cancer

**DOI:** 10.1111/jcmm.15751

**Published:** 2020-09-10

**Authors:** Zhenxing Wang, Yan Zhao, Hongyan Xu, Feihai Liang, Qingxu Zou, Chen Wang, Jingyuan Jiang, Fengwu Lin

**Affiliations:** ^1^ Department of Thoracic Surgery China‐Japan Union Hospital of Jilin University Changchun China; ^2^ Physical Examination Center The Second Hospital of Jilin University Changchun China; ^3^ Department of Medical Oncology The Tumor Hospital of Jilin City Jilin China; ^4^ Department of Cardiovascular thoracic Surgery The Second Affiliated Hospital of Guangxi Medical University Nanning China

**Keywords:** CCL2, CtBP1, NF‐κB, NSCLC, TAMs

## Abstract

The progression of lung cancer is majorly facilitated by TAMs (tumour‐associated macrophages). However, how the TAMs infiltrate the NSCLC microenvironment and the associated biochemical are not fully elaborated. Research has revealed that changes in CtBP1 modulates innate immunity. Here, we investigated if CtBP1 facilitates infiltration of TAM and the subsequent progression of NSCLC. Immunohistochemical analysis was carried out in 96 NSCLC patients to estimate the clinicopathological importance of CtBP1 in the disease. CtBP1 overexpression and knockdown were carried out to assess the activity of CtBP1 in NSCLC cells. Elevated expression of CtBP1 correlated positively with TAMs infiltration into NSCLC tissues, induced EMT (epithelial‐mesenchymal transition) in NSCLC cells and modulated the activated NF‐κB signalling pathway leading to increase in CCL2 secretion from NSCLC cells, thus promoting TAM recruitment and polarization. TAM induction and polarization reduced significantly on exhausting p65 in NSCLC cells with CtBP1. Moreover, infiltration of TMAs was reduced remarkably on antagonist‐mediated blocking of CCR2 and impeded the progression of NSCLC in a mouse model. These findings thus show a novel insight into the process of CtBP1‐regulated TAM infiltration in NSCLC.

## 1 INTRODUCTION

Lung cancer is the leading cause of cancer‐associated human death worldwide, and its five‐year survival rate is only 18%.^1^, ^2^ Non–small‐cell lung cancer (NSCLC) accounts for the largest proportion of lung cancer and is the leading cause of cancer‐related mortality worldwide.^2^, ^3^, ^4^ Studies have revealed the interaction between tumour microenvironment and NSCLC cells and their vital role in progression of NSCLC.^5^, ^6^ Revealing the underlining system of cooperation between components of tumour microenvironment and NSCLC cells may aid in the formulating novel therapeutic strategies and targets.

The most plentiful constituents of tumour physiological environment are TAMs (tumour‐associated macrophages).^7^ The TAMs are derived from myelomonocytic cells and are inducted to the microenvironment of the tumour by tumour‐derived chemokines and cytokines, including VEGF (vascular endothelial growth factor), CCL2 (chemokine C‐C motif ligand 2), M‐CSF (macrophage colony‐stimulating factor) and TGF‐β (transforming growth factor‐beta).^8^, ^9^, ^10^ TAMs are discerned in the tumour microenvironment as alternatively activated macrophages, also known as M2, which are characteristically weak in antigen presentation, marked cytokine and chemokine (such as TGF‐β, interleukin‐10, CCL17 and CCL22) expression.^11^ TAMs promote immune suppression angiogenesis, and metastasis in cancers via the release of chemokines, cytokines, matrix metalloproteases and growth factors. Enhanced infiltration of TAM is related to a poor prognosis in NSCLC.^12^, ^13^ Earlier research has revealed that STAT‐3, NF‐κB (nuclear factor‐κB) and hypoxia‐inducible factor‐1 signalling pathways play a role in recruitment and polarization of TAMs.^14^, ^15^ However, the process involved in TAMs infiltration of the NSCLC cells has not been well elucidated.

Various important cellular processes are modulated by CtBP family proteins.^16^, ^17^ While only a single CtBP protein is expressed in the invertebrate genomes, two related proteins CtBP1 and CtBP2 are coded by the vertebrate genomes, which carry out genetically concomitant and specific roles through the course of development.^18^ CtBP1 and CtBP2 in their primary splice forms act as transcriptional corepressors, whereas the other splice variants have diverse roles in the cytosol.^17^, ^19^ Previous study has shown that CtBP1 interacts with SOX2 to promote the growth, migration and invasion of lung adenocarcinoma.^20^ However, the role of CtBP1 in NSCLC remains to be explored.

In the current study, we evaluated the effect of CtBP1 on recruitment as well as polarization of TAMs by in vitro cell coculture assay and through in vivo animal model studies. The revelations of this study confer new insight into the processes involved in the crosstalk between TAMs and NSCLC cells.

## 2 MATERIALS AND METHODS

### 2.1 Patients and tissue samples

Human NSCLC tissues (n = 96) and paired normal lung tissues (5 cm from the site of tumour) that were healthy were obtained from patients who underwent resection between January 2012 and December 2017 at the China‐Japan Union Hospital of Jilin University. The China‐Japan Union Hospital of Jilin University Research Ethical Committee gave ethical consent towards the use of human samples. Prior to surgery, no patient had undergone any adjuvant therapy. Two pathologists diagnosed the cases independently. The clinical and pathological features of 96 NSCLC patients are presented in detail in Table 1.

**TABLE 1:** Correlation between CtBP1 and clinicopathologic characteristics in patients with NSCLC

Characteristic	No.	CtBP1		p
		Low (n = 48)	High (n = 48)	
Age (years)				
≥60	56	29	27	0.431
<60	40	19	21	
Gender				
Male	45	25	20	0.237
Female	51	23	28	
TNM stage				
I‐II	40	32	8	0.021
III/IV	56	16	40	
Lymph metastasis				
No	33	27	6	0.032
Yes	63	21	42	
Differentiation				
Well	43	19	24	0.109
Moderate/poor	53	29	24	

### 2.2 Cell culture

NSCLC cell lines A549, H460, H1299, H3255, H1975, HCC827, normal immortalized lung epithelial cells HBE and NL20 and human THP‐1 monocytic cells were procured from ATCC (American Type Culture Collection, VA, USA) and grown in RPMI‐1640 medium from Invitrogen (Carlsbad, CA, USA) containing 10% foetal bovine serum (FBS; Hyclone, Logan, UT, USA). All cell lines were grown at 37℃ in an incubator containing 5% CO_2_ and appropriate humidity. THP‐1 monocytes differentiated into macrophages (as THP‐1 macrophages) after one full day incubation in the presence of 150 nM phorbol 12‐myristate 13‐acetate (P1585), from Sigma‐Aldrich (St Louis, MO, USA).

A549 and H1299 cells which stably overexpressed CtBP1, and A549 and H1299 cells with CtBP1 knockdown were confirmed. To develop NSCLC CM, NSCLC cells at 5 × 10^6^/mL with varying treatments were given three washes with serum‐free RPMI1640 and further maintained in serum‐free RPMI‐1640 for next three full days. The supernatant was collected after filtering through 0.22 μm filters and kept at 4℃ until further studies.

### 2.3 Immunohistochemistry

EnVision™ Flex + System (Dako, Santa Clara, CA, USA) was used to stain the lung cancer sections that were fixed in paraformalin and embedded in paraffin. EnVision™ Flex target retrieval solution (same brand) was employed to deparaffinize and unmask epitopes in the samples. Endogenous peroxidase was blocked by adding 0.3% hydrogen peroxide for 5 min. This was followed by the addition of primary antibody at 4°C overnight. Subsequently, the following treatments were done: 15 min with EnVision™ Flex linker rabbit, 30 min with EnVision™ Flex/HRP enzyme and 10 min with 3′‐3‐diaminobenzidine tetrahydrochloride. Haematoxylin was added as a counterstain followed by dehydration and mounting on a Richard‐Allan Scientific Cytoseal XYL (Thermo Scientific, Waltham, MA, USA). The cells that stained positive were evaluated at × 400 magnification in a minimum of five areas and based on the scores were categorized per specimen as: score 0, negative; score 1, <25% positive cells; score 2, 25%‐50% positive cells; score 3, 50%‐75% positive cells; and score 4, >75% positive cells. This was followed by scoring of immunostaining intensity as follows: 1+, weak; 2+, moderate; and 3+, intense. A total score was obtained by multiplying the two above‐mentioned values. A low expression was indicated by a total score ≤4 and >4 indicated a high expression. Three experienced investigators blinded to the patient conditions evaluated the immunostaining independently.

### 2.4 Migration assay

Macrophage migration induced by CM from NSCLC cells after varying treatments was evaluated on Transwell plates (24‐well; Corning Inc, NY, USA). To the top chamber of transwell plates, the collected THP‐1 macrophages were added. Concurrently, at the bottom of the transwell chamber, RPMI‐1640 and CM media with 20% FBS were added. After one full day, the cells that crossed the inserts were counted under phase‐contrast microscopy after crystal violet staining. The average number of inserted cells was determined after randomly selecting five fields.

### 2.5 Enzyme‐linked immunosorbent assay

After various treatments, the NSCLC cells were kept in a medium without serum for two full days and the culture supernatant was collected to measure CCL2 concentration. Next, incubation of THP‐1 macrophages was done with CM for two full days to determine the concentration of IL‐10 as well as CCL17 and CCL22. After giving thrice wash with PBS, the cells were kept in medium with no serum for two full days and the culture supernatant was collected to determine CCL2, IL‐10, CCL17 and CCL22 concentration using ELISA Kit as per instructions.

### 2.6 Real‐time RT‐PCR

The real‐time PCR was performed as previous studies.^21^, ^22^ The RNeasy Mini Kit from Qiagen was used to extract total RNA as per instructions and RNase‐Free DNase set from Qiagen was used to remove the contaminating genomic DNA. Reverse transcription of RNA was done with Invitrogen's M‐MLV Reverse Transcriptase (200 units and random primers (1μg; Amersham Bioscience, USA), dNTP (0.01M each), First‐Strand buffer (1x) and dithiothreitol (0.1 M) after heat deactivation. The cDNA was amplified through qPCR using specific primers and SYBR Green chemistry on Applied Biosystems 7500 Real‐time PCR system (CA, USA). For all primer sets, same conditions were applied, namely 10 min at 95℃, then 15 s at 95℃ for 40 cycles and 1 min at 60℃ and the fluorescence was measured during the annealing/extension phase. The Sequence Detection System was used to generate amplification plots. The internal control in this assay was GAPDH expression. The primers are list in Table 2.

**TABLE 2:** Primers list

	Forward	Reverse
CtBP1	5'‐CGACCTCCGATCATGAAC‐3'	5'‐GCTAAAGCTGAAGGGTTCC‐3'
CCL2	5'‐AGGTCCCTGTCATGCTTCTG‐3'	5'‐GGGATCATCTTGCTGGTGAA‐3'
CSF‐1	5'‐GCACCAACAACGCTACCT‐3'	5'‐CGAACACGTCCACCTCCT‐3'
CCL5	5'‐TGCAGAGGACTCTGAGACAGC‐3'	5'‐GAGTGGTGTCCGAGCCATA‐3'
IL‐8	5'‐AAACTGGCTGTTGCCTTCTTT‐3'	5'‐ATTTCAGCACTGGCATCGAA‐3'
IL‐4	5'‐CCGTAACAGACATCTTTGCTGCC‐3'	5'‐GAGTGTCCTTCTCATGGTGGCT‐3'
IL‐13	5'‐GAGTGTGTTTGTCACCGTTG‐3'	5'‐TACTCGTTGGTCGAGAGCTG‐3'
CCL20	5'‐CTGGCTGCTTTGATGTCAGT‐3'	5'‐CGTGTGAAGCCCACAATAAA‐3'
CXCL4	5'‐CCCTAGACCCATTTCCTCAA‐3'	5'‐AGAAACAACAGGCCCAGAAG‐3'
CCL7	5'‐CTGCTCTCCAGCGCTCTCA‐3'	5'‐GTAAGAAAAGCAGCAGGCGG‐3'
IL‐6	5'‐TGTCTATACCACTTCACAAGTCGGAG‐3'	5'‐GCACAACTCTTTTCTCATTTCCAC‐3'
CCL18	5'‐CCCCAAGCCAGGTGTCATCCTC‐3'	5'‐GGGCCATTGCCCTGGCTCAG‐3'
TNF‐α	5'‐GACCCTCACACTCAGATCATCTTCT‐3'	5'‐CCACTTGGTGGTTTGCTAGGA‐3'
TGF‐β	5'‐TATCGACATGGAGCTGGTGAAG‐3'	5'‐CAGCTTGGACAGGATCTGGC‐3'
VEGF	5'‐AAGCCAGCACATAGGAGAGATGA‐3'	5'‐TCTTTCTTTGGTCTGCATTCACA‐3'
CXCL5	5'‐GCCCTACGGTGGAAGTCATA‐3'	5'‐AGTGCTTCCGCTTAGCTTT‐3'
CXCL2	5'‐GCTACAGGGGCTGTTGTGGCC‐3'	5'‐CAGGCTCCTCCTTTCCAGGTCA‐3'
CXCL13	5'‐GGGTGCCCAAAAAGAGAAATC‐3'	5'‐GATGGGAGGGTTCAAGCATACA‐3'
CD163	5'‐CAGGAAACCAGTCCCAAACA‐3'	5'‐AGCGACCTCCTCCATTTACC‐3'
CD86	5'‐CTGCTCATCTATACACGGTTACC‐3'	5'‐GGAAACGTCGTACAGTTCTGTGG‐3'
IL‐10	5'‐ATGCCCCAAGCTGAGAACCA‐3'	5'‐AAGGGGCTGGGTCAGCTATCCCA‐3'
CCL22	5'‐ATGGATTGCCTGAGCCTG‐3'	5'‐CCTTTGTGGTCCCATATTCTGTC‐3'
CCL17	5'‐ACATTGCTTTTCCCCTTTGAGCCT‐3'	5'‐GACTTTTCTGCAGCAGTGCCAG‐3'
GAPDH	5'‐CAATGACCCCTTCATTGACC‐3'	5'‐GACAAGCTTCCCGTTCTCAG‐3'

### 2.7 Western blotting

The Western blotting was performed as previous studies.^23^, ^24^ Briefly, cell samples were mixed with lysis buffer (0.01 M Tris‐HCl, pH 7.4; KCl, 0.15M; NaF, 0.1M; EDTA, 0.002M; β‐mercaptoethanol, 0.012 M; Nonidet P‐40, 0.5%) and lysed for 2 hours at 4℃ in the presence of a mixture of protease inhibitors (leupeptin, 0.01 mg/mL; Na_3_VO_4,_ 0.001M; Pefabloc, 0.3 mg/mL; okadaic acid, 0.01 μM). The proteins were mixed with sample buffer to denature and heated at 95°C for 1 min, separated through SDS‐PAGE (10% polyacrylamide gel) and transferred onto membranes of PVDF. Membranes were blocked with fat‐free milk (5% in TBST: 50 mM Tris/pH 7.5, containing 0.15M NaCl and 0.05% Tween‐20) for 60 min at room temperature and probed overnight with primary antibodies at 4°C. The blots were washed, kept in the secondary antibody for 60 min at room temperature, the bands observed and evaluated through UVP Bio Imaging systems. The primary antibodies used are as list: CtBP1 (ab129181, Abcam, Cambridge, MA, USA), N‐cadherin (ab76011, Abcam), E‐cadherin (ab15148, Abcam), Vimentin (#5741, Cell Signaling Technology, Danvers, MA, USA), CCL2 (@2027, Cell Signaling Technology), p65 (#8242, Cell Signaling Technology), p‐p65 (#3033, Cell Signaling Technology), Lamin A/C (#4777, Cell Signaling Technology) and β‐actin (A5441, Sigma, St. Louis, MO, USA).

### 2.8 Flow cytometry assay

Post‐incubation with different CM for two full days, collection of THP‐1 macrophages was done. The cells were given cold PBS (containing 5% human serum and 0.1% NaN_3_) wash thrice, kept in CD163 antibody conjugated with PE (Phycoerythrin) for 60 min and evaluated through Cytomics FC500 from Beckman Coulter (Fullerton, CA).

### 2.9 Mouse model

The ethics committee of China‐Japan union hospital of Jilin university gave consent to the animal studies. C57BL/6 mice (4‐ to 6‐week‐old female) were categorized into six groups (n = 6/group) and kept in Central Animal Laboratory of China‐Japan union hospital of Jilin university. Transfected LLC2 cells (5 × 10^6^) were subcutaneously injected in the upper back of the mice, tumour diameters and mice weights were measured. The formula: 1/2 (length × width^2^) was used to determine tumour volumes. The ARRIVE (Animal Research: Reporting In Vivo Experiments) guidelines were followed for experimental procedures and animal welfare. No animals were excluded in this study, as no abnormality was observed in weight or any apparent disease symptoms in the mice prior to conducting the experiments. Concurrently, mice were given intraperitoneal injection of 50 mg/kg CCR2 antagonist (every two days for 5 times) or Clodronate‐liposome (200μl/mice, one day prior to cells injection, and then treated the mice one time per week for 2 times), for the antagonist treatment each day after implantation of tumour cells. 19 days post‐implantation, all mice were exterminated. The weight of tumour was measured, and after measuring the tumour weight, tumour tissue samples were assessed by immunohistochemical staining.

### 2.10 Statistical studies

GraphPad Prism 6.0 from GraphPad Software was used for statistical analyses. The results were expressed as the mean ± SD (standard deviation). Two‐tailed independent Student's *t* tests and ANOVA (analysis of variance) were used to analyse quantitative data. Fisher's exact tests and chi‐square tests were used to compare categorical variables. Clinical correlations were analysed using chi‐square (χ^2^) tests, and survival analysis was conducted by the Kaplan‐Meier method along with log‐rank tests. Statistically significant differences were considered when *P* values were < 0.05.

## 3 RESULTS

### 3.1 CtBP1 levels increased and associated with a poor prognosis in NSCLC patients

**FIGURE 1:**
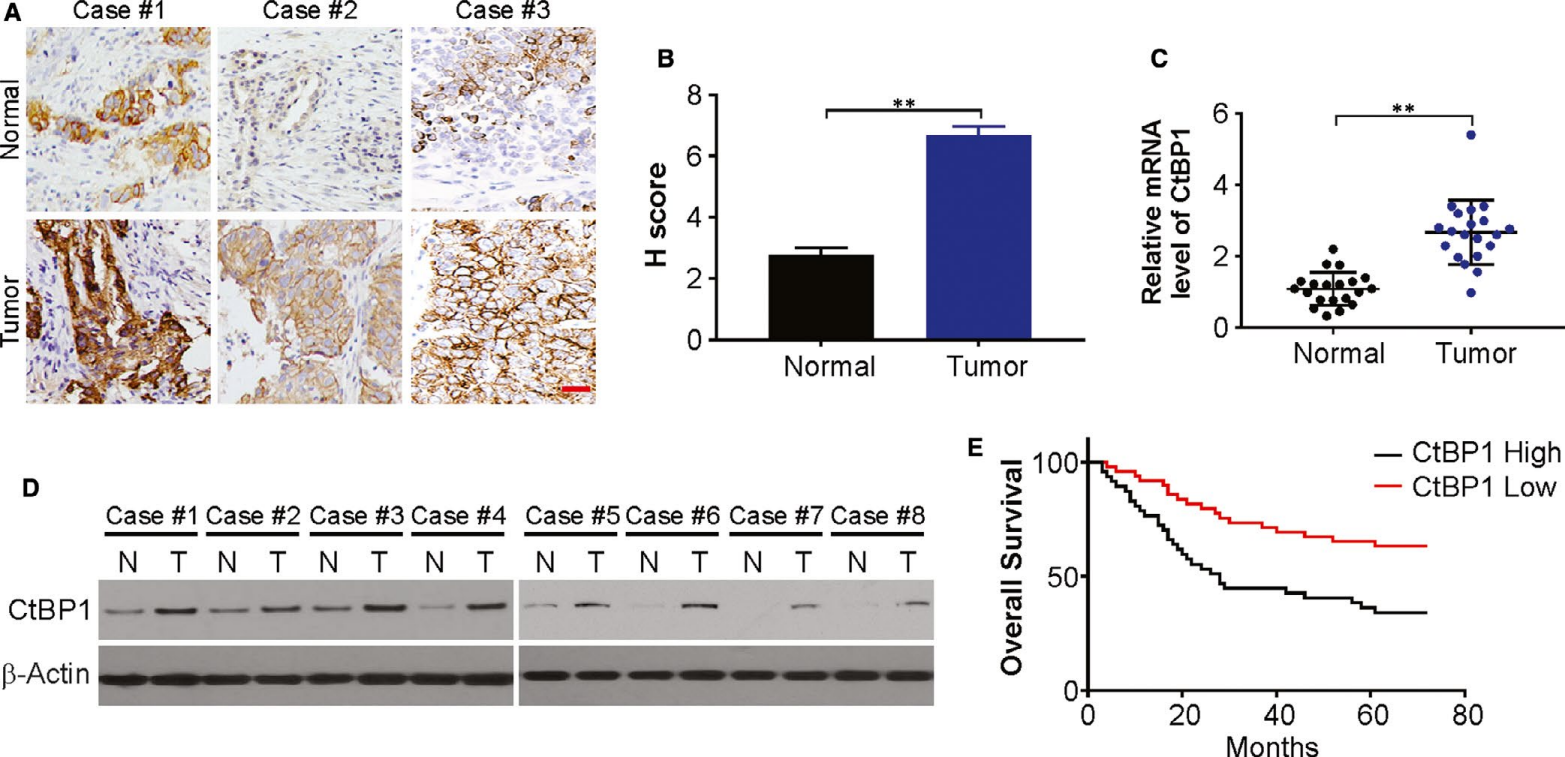
The expression and clinical significance of CtBP1 in NSCLC. (A) Representative images of IHC staining of CtBP1 expression in 96 human NSCLC tissues and pair‐matched normal tissues. Scale bar, 25 μm. (B) H score of IHC staining of NSCLC tissues and pair‐matched normal tissues. (C) Relative mRNA level of CtBP1 in NSCLC samples and pair‐matched normal tissues was analysed by real‐time PCR. (D) Protein level of CtBP1 in NSCLC samples and pair‐matched normal tissues was analysed by Western blotting. (E) Kaplan‐Meier analysis showing the correlations between CtBP1 expression and the overall survival of patients with NSCLC, determined by log‐rank test. CtBP1 low, n = 48; CtBP1 high, n = 48 (*P* = 0.023). Data are derived from three independent experiments and presented as mean ± SD. **P < 0.01

To investigate the role of CtBP1 in NSCLC, the clinical role of CtBP1 was explored in NSCLC samples. Firstly, we evaluated CtBP1 expression using immunohistochemistry (IHC), western blotting, and real‐time PCR assays in NSCLC and paired normal tissues. CtBP1 expression enhanced significantly in NSCLC tissues than in matched, normal tissues, as observed by IHC assay (Figure 1A,B). This up‐regulation was confirmed further in eight paired NSCLC tissues and normal tissues at the transcript and protein levels (Figure 1C,D). We also investigated if there was any correlation between CtBP1 expression with clinical and pathological characteristics and human NSCLC prognosis. Notably, enhanced CtBP1 expression is associated remarkably with TNM in its advanced stage, metastasis of the lymph node, and a poor differentiation of tumour in NSCLC (Table 1). Furthermore, patients with high expression of CtBP1 exhibited a shorter OS (overall survival) than those with low CtBP1, as assessed by Kaplan‐Meier analysis (Figure 1E). Cox's multivariate analysis indicated that this enhanced expression of CtBP1 was deemed an independent risk factor for the prognosis of OS in NSCLC patients (Table 3). Thus, CtBP1 levels up‐regulate frequently in human NSCLC and is related relatively poor survival.

**TABLE 3:** Univariate and multivariate analysis for predictors of overall survival

Variables	Overall survival					
	Univariate analysis			Multivariate analysis		
	HR	95%CI	*P*	HR	95%CI	*P*
Age (<60 vs ≥ 60)	1.013	0.612‐1.672	0.933			
Gender (male vs female)	0.982	0.452‐1.342	0.498			
Lymph node metastasis (no vs yes)	9.092	4.582‐19.029	<0.001	2.982	0.982‐4.302	0.032
TNM stage (I/II vs III/IV)	17.339	7.829‐42.039	<0.001	8.92	4.039‐19.029	0.002
Differentiation (well vs moderate/poor)	8.929	4.292‐17.029	0.058	3.442	1.092‐6.076	0.063
CtBP1 expression (low vs high)	0.092	0.022‐2.019	<0.001	0.298	0.098‐0.682	0.002

### 3.2 CtBP1 promoted the proliferation, migration and invasion of NSCLC cells

**FIGURE 2:**
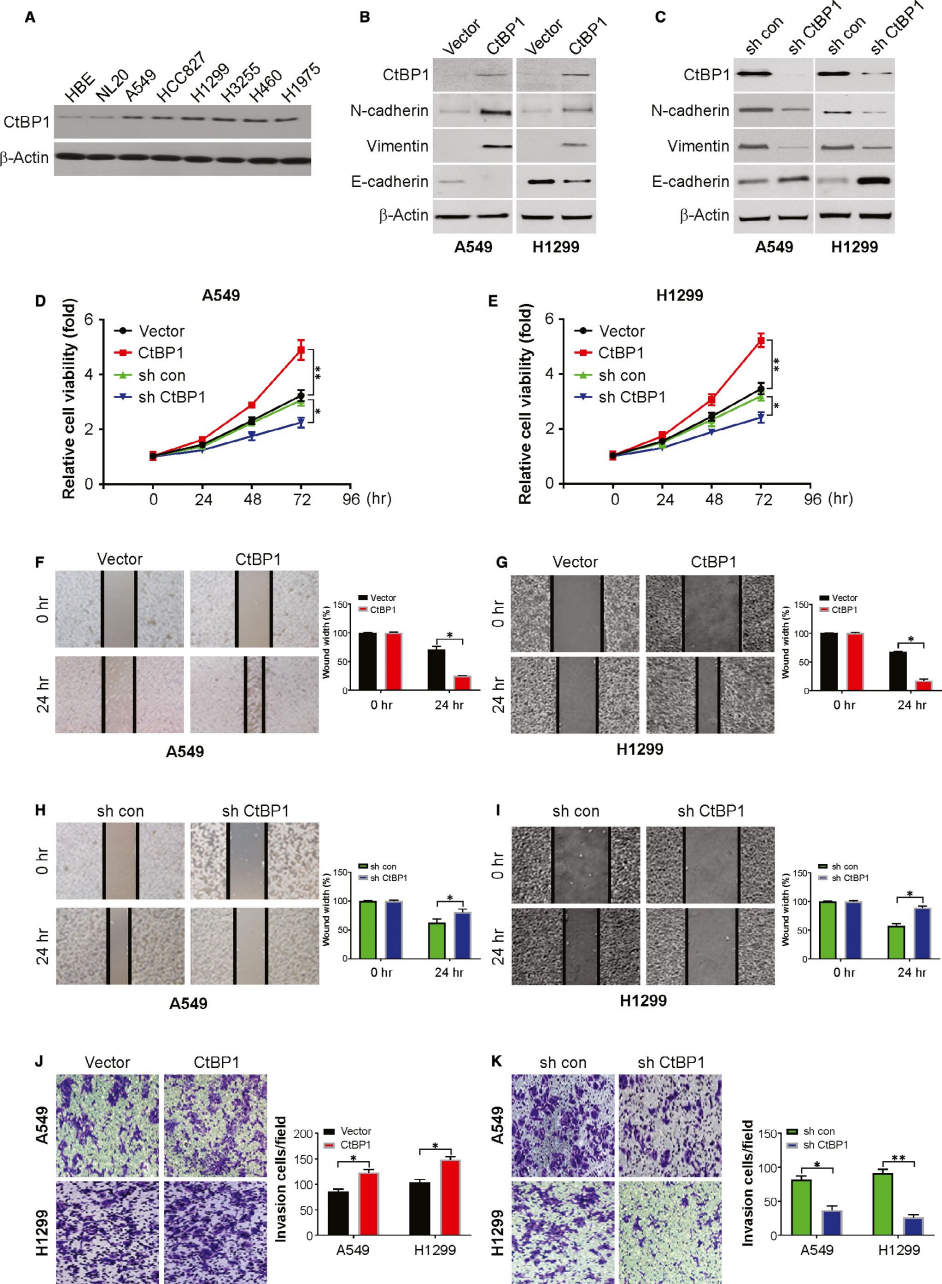
CtBP1 regulates the proliferation, migration and invasion of NSCLC cells in vitro. (A) CtBP1 level in indicated cell lines was analysed by Western blotting. (B) Indicated protein level in control or CtBP1 overexpression cells was analysed by Western blotting. (C) Indicated protein level in sh con or sh *CtBP1* cells was analysed by Western blotting. (D) Cell viability of indicated A549 cells was analysed by CCK‐8. (E) Cell viability of indicated H1299 cells was analysed by CCK‐8. (F) Migration of indicated cells was analysed by wound healing assay. (G) Migration of indicated cells was analysed by wound healing assay. (H) Migration of indicated cells was analysed by wound healing assay. (I) Migration of indicated cells was analysed by wound healing assay. (J) Transwell assays were performed to measure the invasion ability of NSCLC cells. (K) Transwell assays were performed to measure the invasion ability of NSCLC cells. Data are derived from three independent experiments and presented as mean ± SD. *P < 0.05, **P < 0.01

To determine the effect of CtBP1 on the biological characteristics of NSCLC, CtBP1 expression in 6 NSCLC cell lines was evaluated and observed increased CtBP1 expression in all cell lines (Figure 2A). For subsequent studies, H1299 and A549 cells were chosen. Then, stable ectopic CtBP1 expression or knockdown (KD) NSCLC cell lines (A549 and H1299 cells) were established as seen in Figure 2B,C. Overexpression of CtBP1 induced EMT‐like features in terms of protein profiles, including repressed E‐cadherin and enhanced of N‐cadherin and vimentin expression (Figure 2B). However, knockdown of CtBP1 has an opposite effect (Figure 2C). Moreover, there was a marked enhancement in cell proliferation, invasion and migration in the CtBP1 overexpression cells compared with the control cells (Figure 2D‐G,J). In addition, compare with the control knockdown group, knockdown CtBP1 decreased cell proliferation, invasion and migration in A549 and H1299 cells (Figure 2D, E, H, I and K). Thus, our finding demonstrated that CtBP1 could promote in vitro proliferation, invasion and migration of tumour cells.

### 3.3 Elevated CtBP1 levels promote CCL2 secretion from NSCLC cells

Earlier studies report that cytokines play a vital part in remodelling TME and promoting EMT.^25^, ^26^ The cytokines CCL (C‐C motif chemokine ligand)‐2 and −5 promote TAMs recruitment, as well as IL‐8, which is a ligand for C‐X‐C motif CXCR (chemokine receptor)‐1 and −2, are expressed highly on M2 macrophage surface, and TAMs exhibit phenotype like that of M2.^26^ Notwithstanding, alterations induced by CtBP1 in cytokines are not yet extensively understood.

**FIGURE 3:**
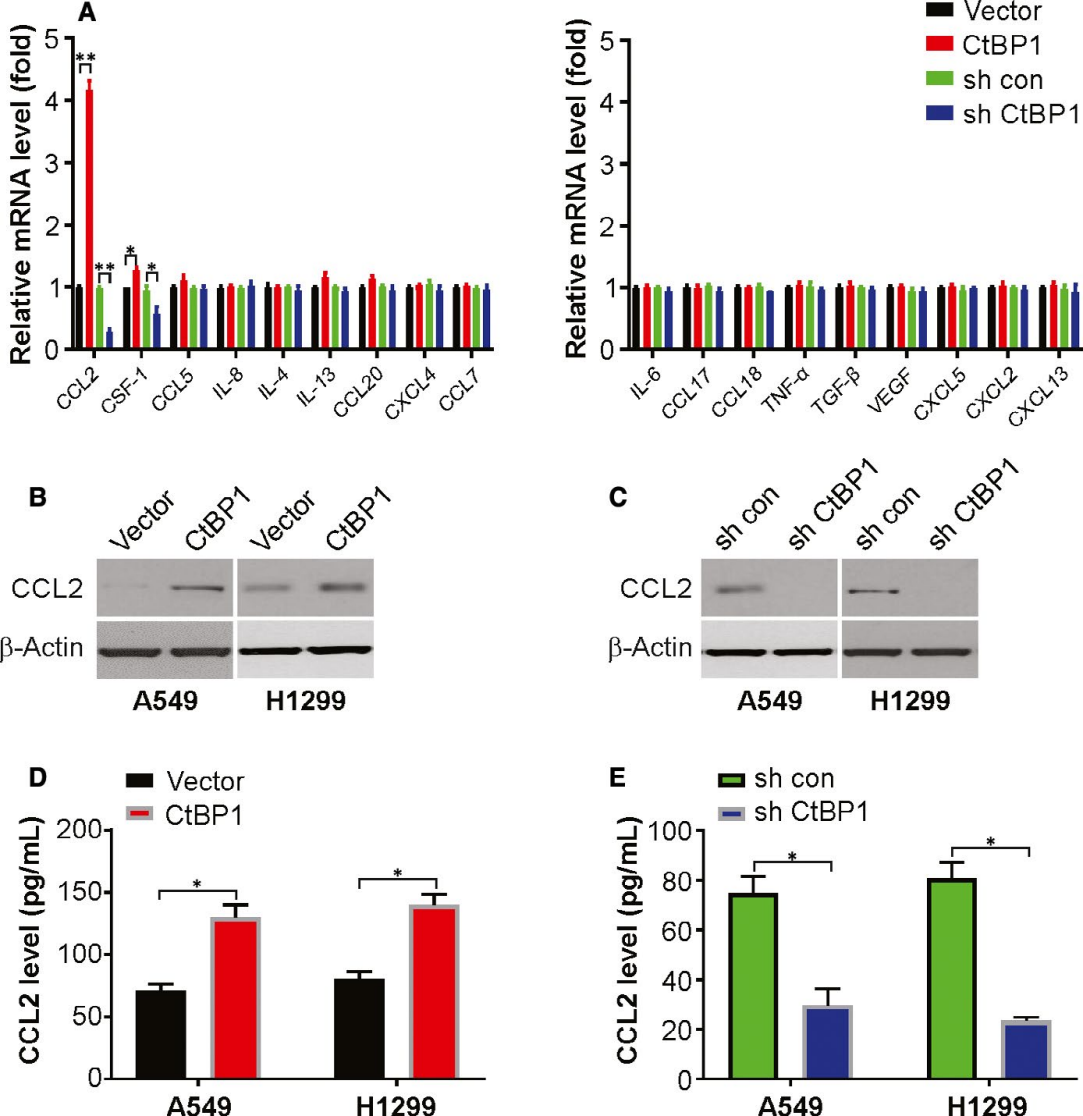
CtBP1 promotes CCL2 induction. (A) Relative mRNA level of indicated cytokines in CtBP1 overexpression or knockdown A549 cells was analysed by real‐time PCR. (B) Protein level of CCL2 in CtBP1 overexpression cells was analysed by Western blotting. (C) Protein level of CCL2 in CtBP1 knockdown cells was analysed by Western blotting. (D) Protein level of CCL2 in CtBP1 overexpression cells was analysed by ELISA. (E) Protein level of CCL2 in CtBP1 knockdown cells was analysed by ELISA. Data are derived from three independent experiments and presented as mean ± SD. *P < 0.05, **P < 0.01

The major cytokines regulated by CtBP1 were found on exploring a series of cytokines and by investigating if CtBP1 could induce the secretion of cytokines to affect the TME (Figure 3A). Indeed, the expression of CCL2 elevated in overexpressing CtBP1 in NSCLC cells (Figure 3B). However, the level of CCL2 decreased in CtBP1 knockdown cells (Figure 3C). Furthermore, ELISA was used for CCL2 secretion analysis. Our findings demonstrated that CtBP1 overexpression promotes CCL2 level, while knockdown CtBP1 decreases CCL2 level (Figurer 3D and 3E). Therefore, the above data indicate that CtBP1 regulates CCL2 expression and secretion in NSCLC cells.

### 3.4 NF‐κB mediated CtBP1‐induced CCL2 up‐regulation

**FIGURE 4:**
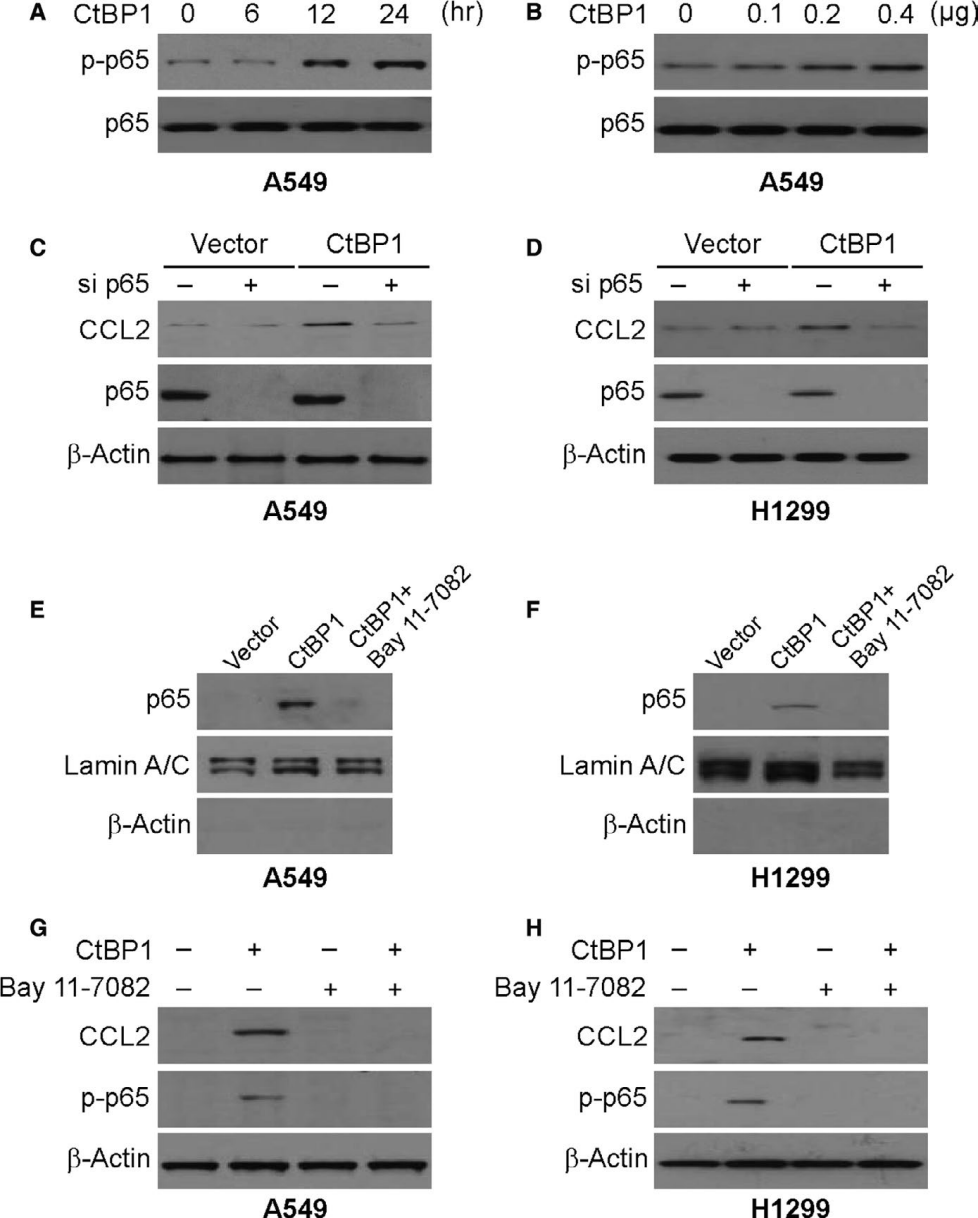
p65 activation is required for CtBP1‐induced CCL2. (A) A549 cells were transfected with 0.4μg CtBP1. Protein was collected at indicated time points. Expression of p‐p65 (S536) and β‐actin was analysed by Western blotting. (B) A549 cells were transfected with CtBP1 at indicated concentration for 24 hours. Expression of p‐p65 (S536) and β‐actin was analysed by Western blotting. (C) A549 cells were transfected with either a control scrambled siRNA or a p65 siRNA with or without CtBP1 cotransfection for 24 hours. CCL2 expression was analysed by Western blotting. (D) H1299 cells were transfected with either a control scrambled siRNA or a p65 siRNA with or without CtBP1 cotransfection for 24 hours. CCL2 expression was analysed by Western blotting. (E) A549 cells were treated with 10 μmol/L BAY11‐7082 for 1 hour and then transfected with CtBP1 for 24 hours. Nuclear fractions were isolated from cells and analysed for p65 expression by Western blotting. Lamin A/C and β‐actin, which are expressed in nucleus and cytoplasm, respectively, were used as controls for loading and fractionation. (F) H1299 cells were treated with 10 μmol/L BAY11‐7082 for 1 hour and then transfected with CtBP1 for 24 hours. Nuclear fractions were isolated from cells and analysed for p65 expression by Western blotting. Lamin A/C and β‐actin, which are expressed in nucleus and cytoplasm, respectively, were used as controls for loading and fractionation. (G) A549 cells were treated with 10 μmol/L BAY11‐7082 for 1 hour and then transfected with CtBP1 for 24 hours. The levels of p‐p65 (S536) and CCL2 were analysed by Western blotting. (H) H1299 cells were treated with 10 μmol/L BAY11‐7082 for 1 hour, and then transfected with CtBP1 for 24 hours. The levels of p‐p65 (S536) and CCL2 were analysed by Western blotting

Then, the process of CtBP1‐mediated CCL2 induction in NSCLC cells was analysed. Studies have revealed that CtBP1 promotes cytokine expression by regulating NF‐κB pathway.^27^ A characteristic of NF‐κB signalling activation is p65 phosphorylation on several residues followed by its movement to the nucleus to activate target genes transcription. Accordingly, overexpression of CtBP1 induced S536 phosphorylation, a primary regulatory site of p65 in A549 cells, time and dosage dependently (Figure 4A and 4B). CtBP1 overexpression‐mediated induction of CCL2 in A549 and H1299 cells was impeded by knocking down of p65 by siRNA (Figure 4C and 4D). Moreover, the Western blotting assay revealed that overexpression CtBP1 led to p65 nuclear translocation in A549 and H1299 cells (Figure 4E and 4F). To assess the role of NF‐κB CCL2 transcription activation in response to CtBP1 overexpression, cells were pretreated with an NF‐κB inhibitor BAY 11‐7082, that impedes p65 nuclear translocation (Figure 4E and 4F). Indeed, CCL2 expression was impeded by BAY 11‐7082 treatment and phosphorylation of p65 induced by CtBP1 overexpression (Figure 4G and 4H), indicating that p65 nuclear translocation mediates CCL2 induction by CtBP1 overexpression.

### 3.5 CtBP1 promotes recruitment and polarization of macrophages by CCL2 in NSCLC cells

**FIGURE 5:**
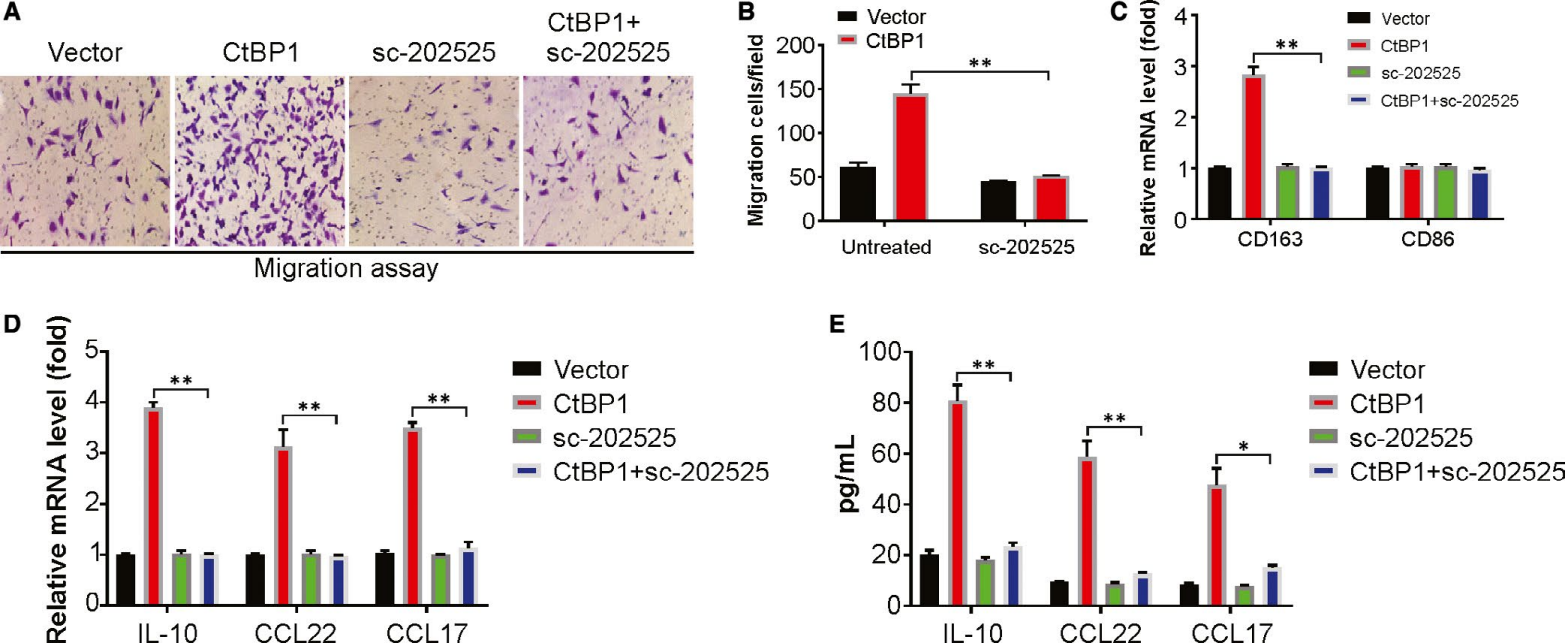
CtBP1 promotes macrophage recruitment and polarization in NSCLC by chemokine (C‐C motif) ligand 2 (CCL2). (A) and (B) Transwell migration assay of macrophage by CM from NSCLC cells as indicated. (C) Real‐time PCR analysis for the expression levels of CD68 and CD163 in THP‐1 macrophages treated with CM from NSCLC cells as indicated. (D) Real‐time PCR for the mRNA expression of tumour‐associated macrophage (TAM) characteristic cytokines in THP‐1 macrophages treated with CM from A549 cells as indicated. (E) Enzyme‐linked immunosorbent assay for the secretion of tumour‐associated macrophage (TAM) characteristic cytokines in THP‐1 macrophages treated with CM from A549 cells as indicated. Data are derived from three independent experiments and presented as mean ± SD. *P < 0.05, **P < 0.01

Several stromal components, including macrophages, play vital parts in progression of a tumour and in TME remodelling.^28^ To investigate whether the enhanced secretion of CCL2 by CtBP1 has a crucial role in infiltration of macrophages, we first carried out an in vitro migration assay and evaluated the effect of CM (conditioned media) of the NSCLC cells with overexpressed CtBP1 on macrophage recruitment ability. Our data indicated that recruitment of macrophages increased significantly in the presence of CM from CtBP1‐overexpressed NSCLC cells in comparison with CM from control cells (Figure 5A,B). Likewise, treatment of macrophages with sc‐202525, a CCR2 antagonist significantly impeded the recruitment effect. Thus, the recruitment of macrophages may be facilitated by the CtBP1‐mediated secretion of CCL2.

Next, the effect of CtBP1 in promoting the polarization of macrophages by CCL2 was evaluated. Treatment of macrophages with CM (from CtBP1‐overexpressed NSCLC cells) led to a remarkable increase transcript of CD163, a TAM marker in comparison with the corresponding control group (Figure 5C). Furthermore, the transcript and protein levels of cytokines characteristic of TAM, such as IL‐10, CCL17 and CCL22 increased significantly in macrophages incubated with CM (from NSCLC cells with overexpressed CtBP1) compared with those in the control group (Figure 5D,E). Thus, the above data shown that CtBP1 possibly promotes macrophage recruitment and polarization by CCL2 in NSCLC.

### 3.6 TAM is required for CtBP1‐induced NSCLC progression

**FIGURE 6:**
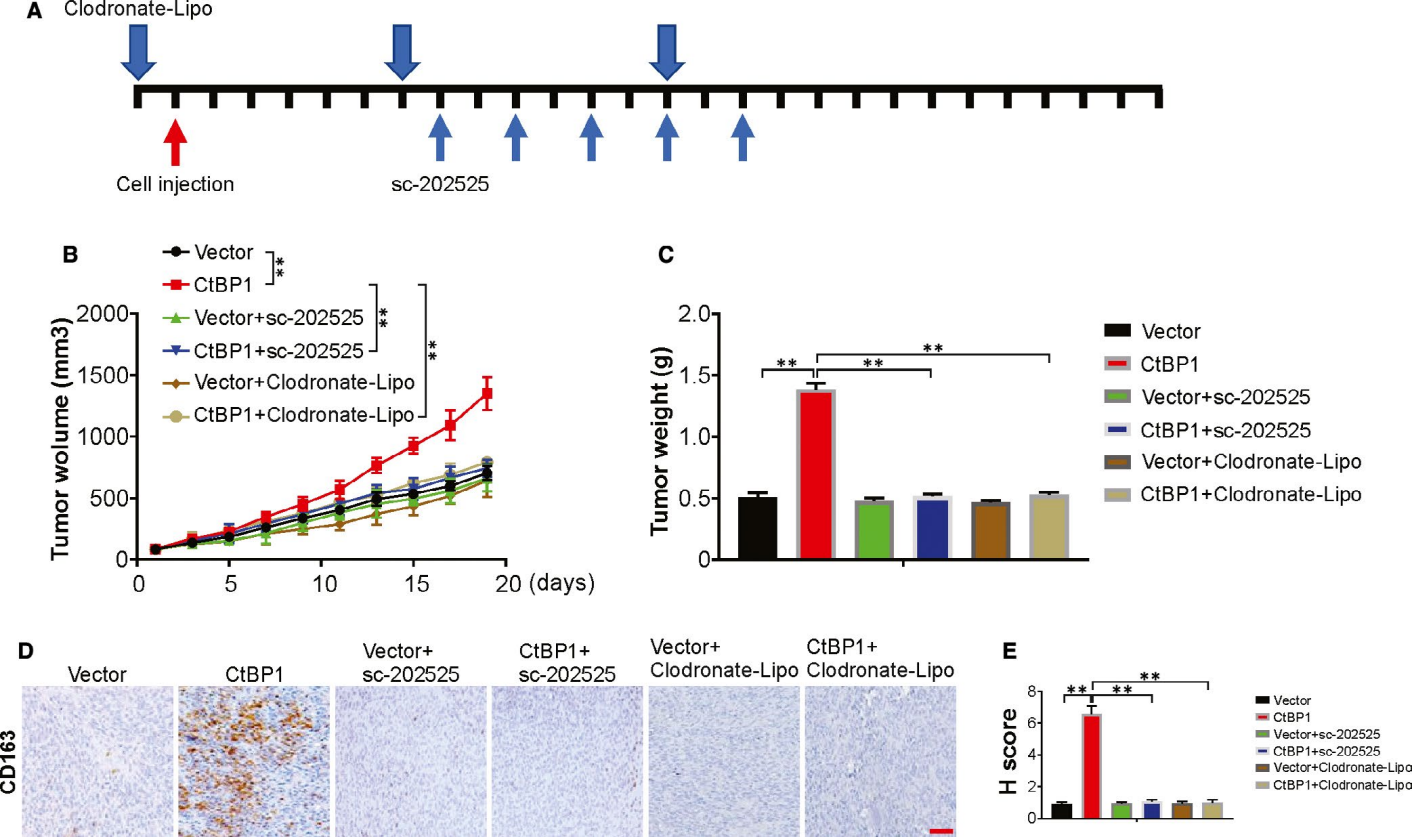
CCL2‐mediated TAM recruitment is required for CtBP1‐induced tumour growth. (A) A schematic view of the treatment plan. (B) Tumour volume of indicated tumours. (C) Tumour weight of indicated tumours. (D) IHC staining of CD163 in indicated tumours. Scale bar, 25 μm. (E) H score of IHC staining of CD163 in tumour tissues. Data are derived from three independent experiments and presented as mean ± SD. **P < 0.01

Finally, the syngeneic NSCLC mice model was used to determine the role of TAM on CtBP1‐mediated NSCLC progression. CtBP1 tumour‐bearing mice were treated with sc‐202525 or Clodronate‐liposome, the tumour growth curve and growth were evaluated. Compared with the control group, overexpression of CtBP1 promotes tumour growth, which was attenuated by sc‐202525 or Clodronate‐liposome treatment (Figure 6A‐6C). To assess the infiltration of TAM, IHC staining was done using human‐specific monoclonal antibody CD163 (10D6). As shown in Figure 6D‐6E, TAM infiltration in tumour microenvironment increased significantly due to overexpression of CtBP1, which could be abrogated by treating with sc‐202525 or Clodronate‐liposome treatment. Thus, these outcomes confirmed that CtBP1 promoted TAM infiltration and progression of NSCLC.

## 4 DISCUSSION

The microenvironment of tumour consists of cytokines, immunocytes and chemokines. The infiltration of lymphocyte happens in glioma and the clinical outcome is predicted by the presence of TILs (tumour‐infiltrating lymphocytes).^29^ Nearly 30% of brain tumour cells consist of macrophages as immunocytes, while 5%‐10% of cells in normal brains are contributed by resident microglia cells.^30^ Within tumour, the M2 cells are primary and help to create a immunosuppressive tumour environment, aid in the growth of glioma cells, angiogenesis and immune escape.^31^ Chemokines and cytokines have a vital part in the development of lung cancer and polarization of M2 cells.^32^ IL‐8 induces M2‐type TAMs and leads to a pro‐oncogenic, inflammatory microenvironment.^33^ The chemokine MIP‐3a (CCL20) may mediate recruitment of macrophages and thus affect tumour growth. A multifunctional cytokine with several functional abilities, IL‐6 is released by M1 macrophages as proinflammatory cytokine and might impede polarization of M2 macrophages.^34^, ^35^

Studies reveal that the crosstalk among TAMs and NSCLC cells in the microenvironment of the tumour has a crucial part in progression of NSCLC.^36^, ^37^ However, the biochemical process involving the interaction between TAMs and NSCLC cells is not yet fully understood. Here, for the first time, we demonstrate that increased CtBP1 promotes the infiltration of TAM and NSCLC progression significantly by activating increased production of CCL2 via the NF‐κB signalling pathway.

The CtBP1/2 is encoded by two analogous genes, CtBP1 and CtBP2, and belongs to the dimeric family of proteins, which have considerable functions in development of animal cells.^17^, ^38^ CtBP form either homo‐ or heterodimers in the presence of NAD (nicotinamide adenine dinucleotide) and can interact with transcriptional factors specific for each gene and recruit several known epigenetic modifying enzymes to the target genes such as HDACs, LSD1, G9a and so on.^38^ Several important tumour suppressor genes were repressed directly by CtBP, which also plays role in the EMT during metastasis of cancer cells and other processes.^39^ CtBP‐target genes were extensively profiled in breast cancer cells, suggesting CtBP as an autonomous factor for initiation, progression and metastasis of tumour by regulating genes at transcriptional level, associated with genome stability, stem cell pathways, EMT and metabolism of cancer cells.^40^

TAMs form a significant part of the tumour microenvironment and have been broadly implicated in NSCLC advancement and infiltration of TAM often correlates with poor prognosis of NSCLC.^10^ The relationship between NSCLC cell‐mitochondrial dynamics and recruitment and polarization of TAM in microenvironment of the tumour has not been elucidated widely. Our study reveals a significantly positive correlation of CtBP1 overexpression with CD163‐positive cell percentage in NSCLC tissues, proposing an obvious link between TAM infiltration and increased CtBP1 levels.

The tumour‐derived chemokines and cytokines, such as CCL2, M‐CSF, VEGF and TGF‐β often recruit TAMs to the tumour microenvironment.^7^ In hypoxic microenvironment, NSCLC cells potentially promote recruitment and polarization of TAMs by cytokine secretion, thus inducing a tumour microenvironment with suppressed immunity and facilitate metastasis.^13^ We observed that elevated levels of CtBP1 in NSCLC cells promoted TAM recruitment and polarization, in accordance with above‐mentioned findings. Our study also showed that CCL2 secretion is induced by enhanced CtBP1, the most important chemoattractant for TAM recruitment and polarization, consistent with previous findings. Being a factor with several functions, CCL2 takes part in diverse aspects of liver disease, such as cirrhosis and hepatocarcinogenesis.^41^ Research shows that the polarization and accumulation of macrophages through CCR2 receptor signalling pathway are aided by CCL2.^42^ Additionally, targeting signalling of CCL2/CCR2 with a CCR2 antagonist was found to remarkably reduce TAM recruitment and polarization, thus enhancing the immunotherapeutic effect of tumour.^42^ Accordingly, our study showed CCL2 as a vital mediator to link CtBP1 and TAM infiltration. This finding was further confirmed in an orthotopic nude NSCLC mice model by injecting antagonist of CCR2.

Being a prototypical inflammatory and immune signalling pathway, NF‐κB pathway is a primary modulator of innate immunity and inflammation.^43^ A NF‐κB subunit, p65, is normally localized by its inhibitor IκB to the cytoplasm.^43^ Translocation of NF‐κB p65 to the nucleus occurs after stimulation and functions as a transcription factor following dissociation of IκB from NF‐κB.^44^ We found in this study that the result of CtBP1 overexpression is CCL2 production and significantly suppressed phosphorylated p65 nuclear translocation as a result of treatment with NF‐κB inhibitor. These observations thus implicate the involvement of signalling pathway for NF‐κB in CCL2 secretion and production induced by CtBP1.

In conclusion, increased CtBP1 level was observed in NSCLC cells, which up‐regulated CCL2 secretion, to aid in TAM recruitment and polarization and subsequently facilitated progression of NSCLC. The outcomes show overexpressed CtBP1‐mediated interaction between NSCLC cells and TAMs. The current study further enhances our knowledge of the process involving function of CtBP1 in NSCLC progression.

## CONFLICT OF INTEREST

The authors declare that they have no conflict of interest.

## AUTHOR CONTRIBUTION

ZW, YZ, HX, FL, QZ, CW and JJ: Experiments. ZW, YZ and FL: Study design. HX, FL and QZ: Data analysis. ZW and FL: Manuscript drafting. All authors: Manuscript reviewing and approval..

## Data Availability

The data used to support the findings of this study are available from the corresponding author upon request.
