# Correction to “CtBP1 promotes tumour‐associated macrophage infiltration and progression in non–small‐cell lung cancer”

**DOI:** 10.1111/jcmm.18189

**Published:** 2024-05-21

Wang Z, Zhao Y, Hongyan X, et al. CtBP1 promotes tumour‐associated macrophage infiltration and progression in non–small‐cell lung cancer. *J Cell Mol Med*. 2020;24:11445‐11456.

In Zhenxing Wang et al.,^1^ some images were misused, and some data need to be supplemented. The relevant details and correct images are shown below. The authors confirm all results and conclusions of this article remain unchanged.

1. The images in Figure 1A,D were misused. In Figure 1B,C, the number of cases was not marked. In Figure 1D, the statistical data of CtBP1 expression were not provided. The corrected Figure 1 is shown below.

**FIGURE 1:**
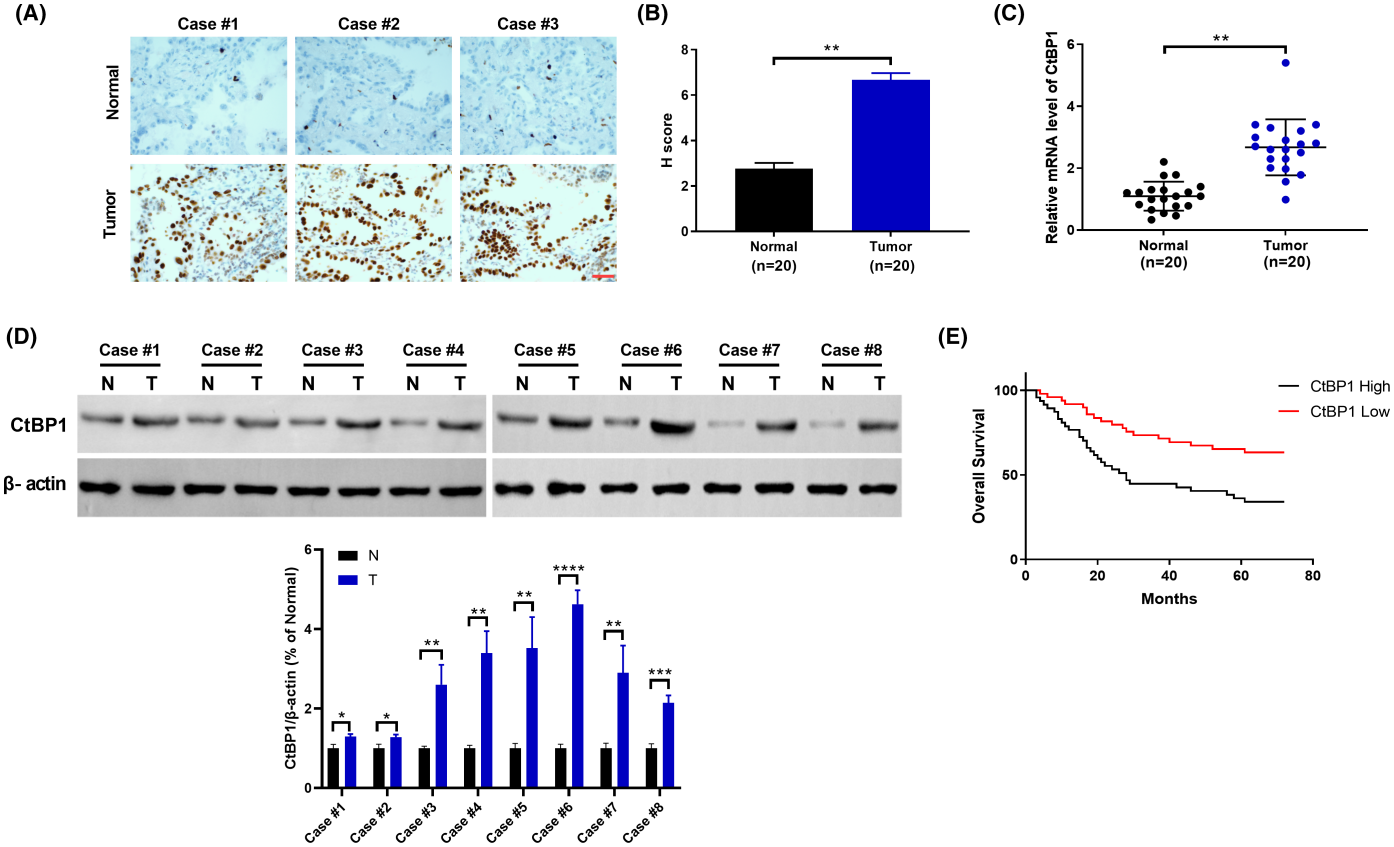
The expression and clinical significance of CtBP1 in non**–**small‐cell lung cancer (NSCLC). (A) Representative IHC staining images of CtBP1 expression in 96 human NSCLC tissues and paired normal tissues. Scale bar, 50 μm. (B) IHC staining H scores for NSCLC tissues and paired normal tissues. (C) Relative CtBP1 mRNA levels in NSCLC samples and paired normal tissues were analysed by real‐time PCR. (D) CtBP1 protein levels in NSCLC samples and paired normal tissues were analysed by Western blotting. (E) Kaplan–Meier analysis showing the correlations between CtBP1 expression and the overall survival of patients with NSCLC, as determined using a log‐rank test. CtBP1 low, *n* = 48; CtBP1 high, *n* = 48 (*p* = 0.023). Data are derived from three independent experiments and presented as mean ± SD. **p < 0.01.

2. Western blotting and transwell images in Figure 2 were misused. The correct images are shown below.

**FIGURE 2:**
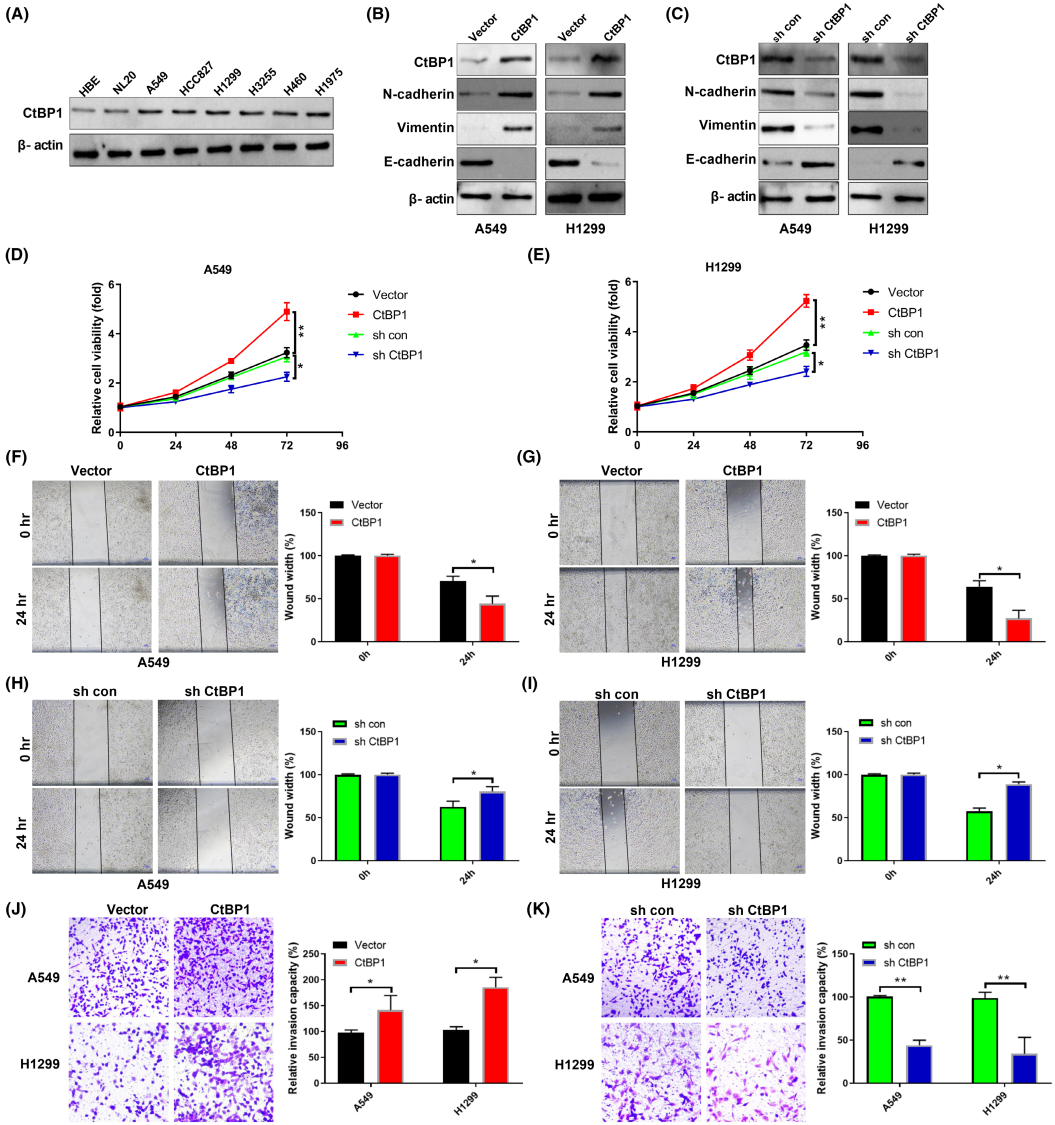
CtBP1 regulates the proliferation, migration and invasion of NSCLC cells in vitro. (A) CtBP1 levels in the indicated cell lines were analysed by Western blotting. (B) Levels of the indicated proteins in control or CtBP1‐overexpressing cells were analysed by Western blotting. (C) Levels of the indicated proteins in shCon or shCtBP1 cells were analysed by Western blotting. (D, E) The viability of the indicated A549 (D) and H1299 (E) cells was analysed by CCK‐8 assay. (F–I) The migration of the indicated cells was analysed by wound healing assay. (J, K) Transwell assays were performed to measure the invasion ability of NSCLC cells. Data are derived from three independent experiments and presented as mean ± SD. *p < 0.05, **p < 0.01.

3. Dividing the results into two groups is not necessary in Figure 3A. The images in Figure 3B,C were misused. The corrected Figure 3 is shown below.

**FIGURE 3:**
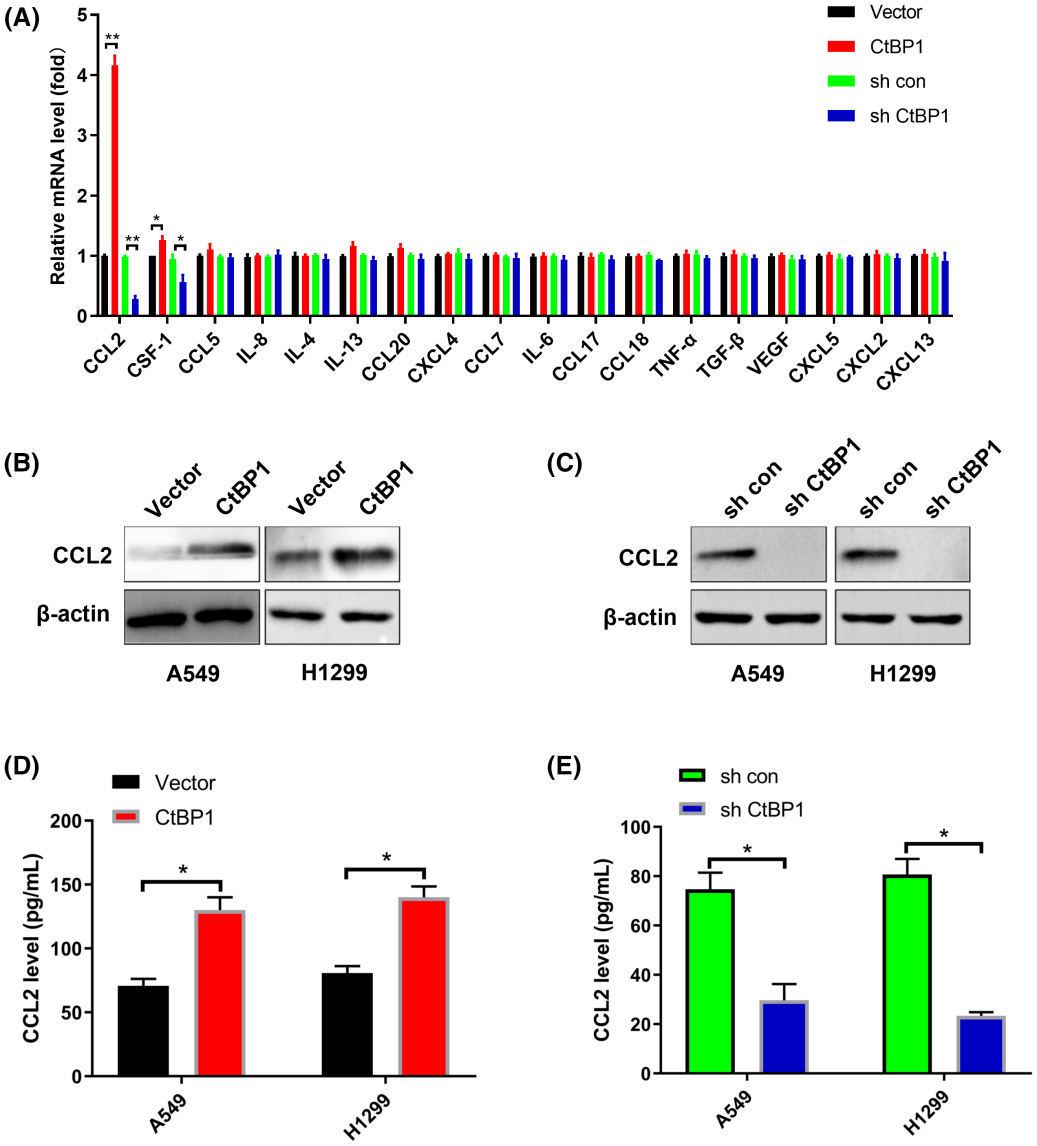
CtBP1 promotes CCL2 induction. (A) Relative mRNA levels of the indicated cytokines in A549 cells in which CtBP1 was overexpressed or knocked down were analysed by real‐time PCR. (B) Protein level of CCL2 in CtBP1‐overexpressing cells was analysed by Western blotting. (C) CCL2 protein levels in CtBP1 knockdown cells were analysed by Western blotting. (D) Protein levels of CCL2 in CtBP1‐overexpressing cells were analysed by ELISA. (E) Protein levels of CCL2 in CtBP1 knockdown cells were analysed by ELISA. Data are derived from three independent experiments and presented as mean ± SD. *p < 0.05, **p < 0.01.

4. Western blotting images in Figure 4 were misused. The correct images are shown below.

**FIGURE 4:**
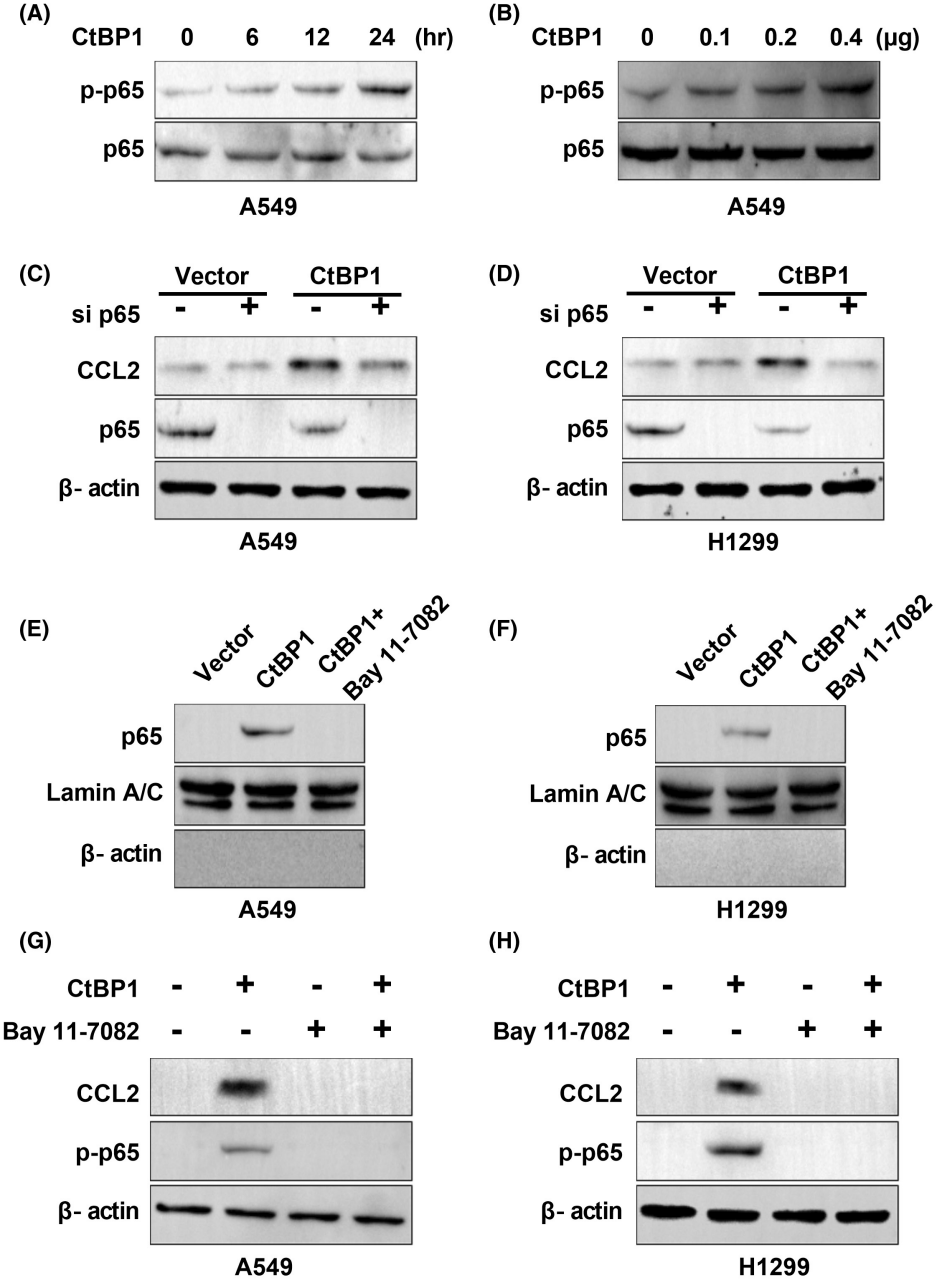
p65 activation is required for CtBP1‐induced CCL2 production. (A) A549 cells were transfected with 0.4 μg CtBP1. Protein was collected at indicated time points. The expression of p‐p65 (S536) and β‐Actin was analysed by Western blotting. (B) A549 cells were transfected with CtBP1 at indicated concentration for 24 h. Expression of p‐p6 (536) and β‐Actin was analysed by Western blotting. (C) A549 cells were transfected with either a control scrambled siRNA or a p65 siRNA with or without CtBP1 co‐transfection for 24 h. CCL2 expression was analysed by Western blotting. (D) H1299 cells were transfected with either a control scrambled siRNA or a p65 siRNA with or without CtBP1 co‐transfection for 24 h. CCL2 expression was analysed by Western blotting. (E) A549 cells were treated with 10 μmol/L MY11‐7082 for 1 h and then transfected with CtBP1 for 24 h. Nuclear fractions were isolated from cells and analysed for p65 expression by Western blotting. Lamin A/C and β‐Actin, which are expressed in the nucleus and cytoplasm, respectively, were used as controls for loading and fractionation. (F) H1299 cells were treated with 10 μmol/L BAY11‐7082 for 1 h and then transfected with CtBP1 for 24 h. Nuclear fractions were isolated from cells and analysed for p65 expression by Western blotting. (G) A549 cells were treated with 10 μmol/L BAY11‐7082 for 1 h and then transfected with CtBP1 for 24 h. The levels of p‐p65 (S536) and CCL2 were analysed by Western blotting. (H) H1299 cells were treated with 10 μmol/L BAY11‐7082 for 1 h and then transfected with CtBP1 for 24 h. The levels of p‐p65 (S536) and CCL2 were analysed by Western blotting.

5. The images in Figure 5A were misused. Some new data need to be supplemented. The corrected Figure 5 is shown below.

**FIGURE 5:**
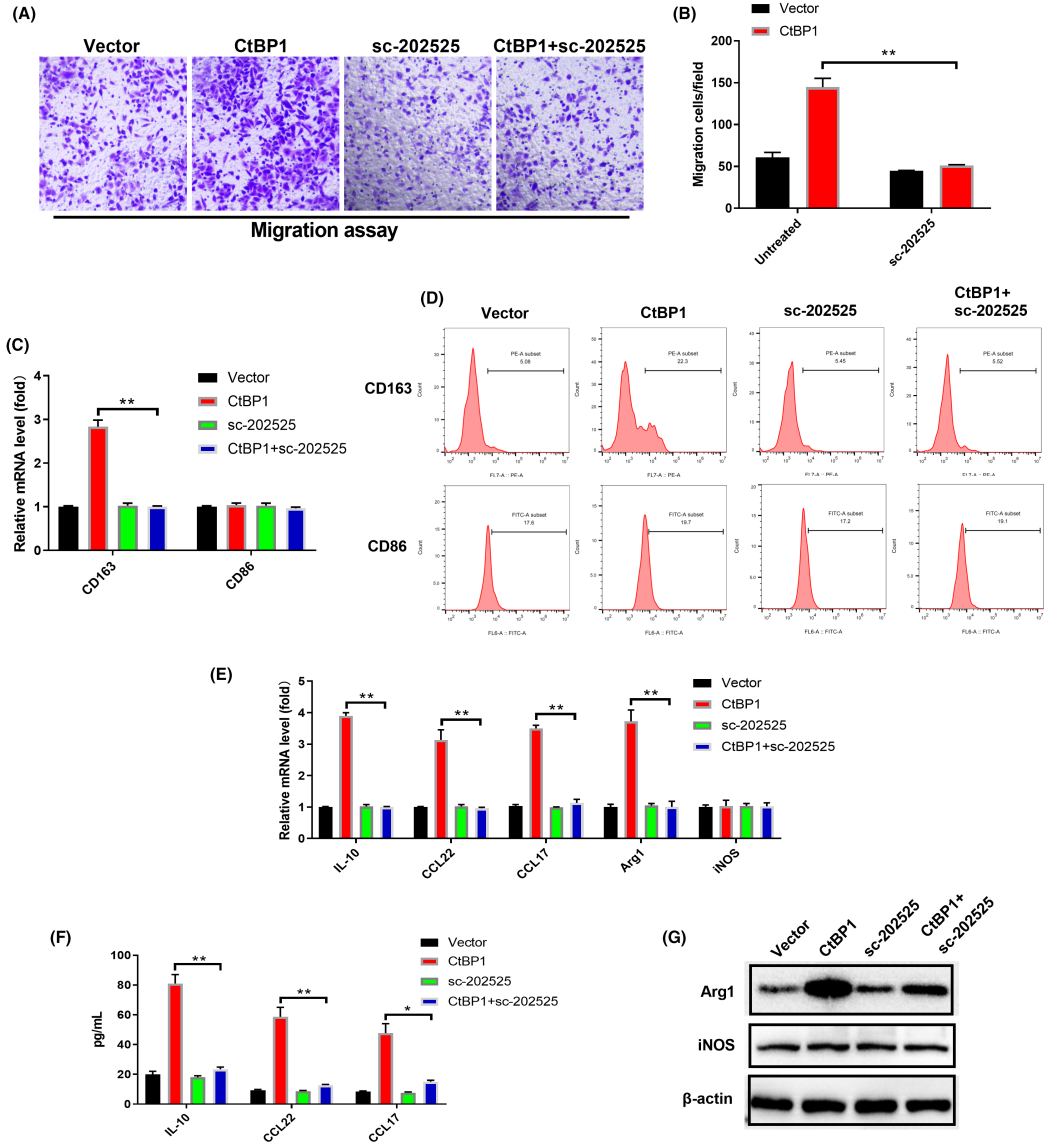
CtBP1 promotes macrophage recruitment and polarization in NSCLC through a mechanism mediated by CCL2. (A, B) Transwell migration assay of macrophages by CM from NSCLC cells as indicated. (C, D) CD68 and CD163 expression in THP‐1 macrophages treated with CM from NSCLC cells as indicated was assessed by real‐time PCR and flow cytometry. (E) Real‐time PCR was used to analyse the mRNA levels of characteristic tumour‐associated macrophage (TAM) cytokines in THP‐1 macrophages treated with CM from A549 cells as indicated. (F, G) ELISAs and Western blotting were used to assess the secretion of characteristic TAM cytokines from THP‐1 macrophages treated with CM from A549 cells as indicated. Data are derived from three independent experiments and presented as the mean ± SD. *p < 0.05, **p < 0.01.

6. The images in Figure 6D were misused. The correct images are shown below.

**FIGURE 6:**
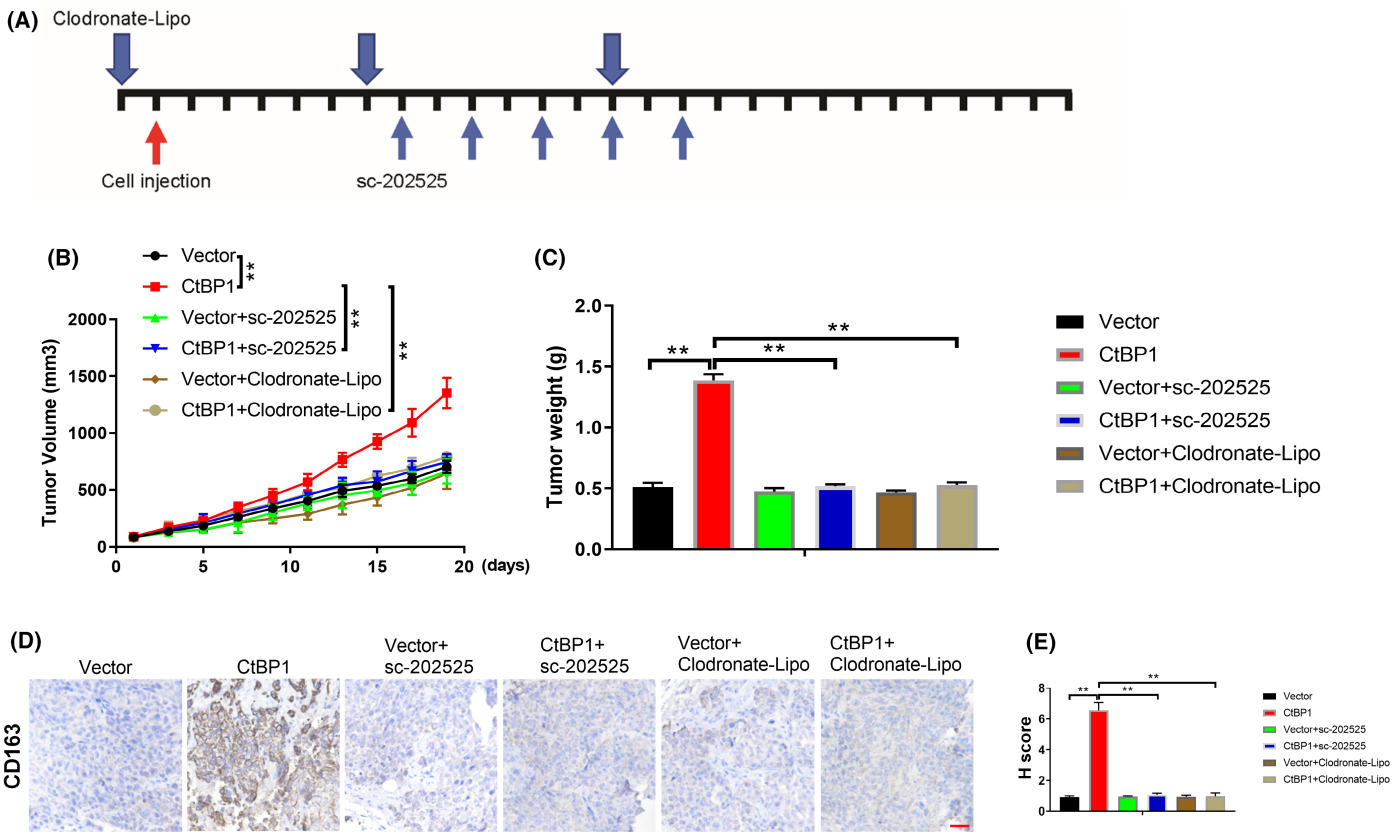
CCL2‐mediated TAM recruitment is required for CtBP1‐induced tumour growth. (A) Schematic overview of the treatment plan. (B) Tumour volumes. (C) Tumour weights. (D) IHC staining for CD163 in the indicated tumours; Scale bar, 25 μm. (E) IHC staining H scores for CD163 in tumour tissues. Data are derived from three independent experiments and presented as the mean ± SD. **p < 0.01.
